# Prescription patterns of granulocyte colony–stimulating factors in patients with breast cancer: A real-world study

**DOI:** 10.1371/journal.pone.0288642

**Published:** 2023-07-17

**Authors:** Shu-Wei Hsu, Shao-Chin Chiang, Jason C. Hsu, Yu Ko

**Affiliations:** 1 Department of Clinical Pharmacy, School of Pharmacy, College of Pharmacy, Taipei Medical University, Taipei, Taiwan; 2 Department of Pharmacy, Cardinal Tien Hospital, New Taipei City, Taiwan; 3 Department of Pharmacy, College of Pharmaceutical Sciences, National Yang Ming Chiao Tung University (Yang Ming Campus), Taipei, Taiwan; 4 Department of Pharmacy, Koo Foundation Sun Yat-Sen Cancer Center, Taipei, Taiwan; 5 International Ph.D. Program in Biotech and Healthcare Management, College of Management, Taipei Medical University, Taipei, Taiwan; 6 Clinical Data Center, Office of Data Science, Taipei Medical University, Taipei, Taiwan; 7 Research Center of Health Care Industry Data Science, College of Management, Taipei Medical University, Taipei, Taiwan; 8 Clinical Big Data Research Center, Taipei Medical University Hospital, Taipei Medical University, Taipei, Taiwan; 9 Research Center for Pharmacoeconomics, College of Pharmacy, Taipei Medical University, Taipei, Taiwan; Tehran University of Medical Sciences, ISLAMIC REPUBLIC OF IRAN

## Abstract

**Background and objectives:**

Myelosuppressive chemotherapy is effective for breast cancer but carries a potential risk of febrile neutropenia (FN). Clinical practice guidelines have recommended prophylaxis with granulocyte colony-stimulating factor (G-CSF) to reduce the incidence of FN in patients receiving chemotherapy. We aimed to examine the use of G-CSFs for primary prophylaxis for FN and to see whether it follows the guidelines. In addition, we examined the changes in the use of long-acting and short-acting G-CSFs in patients with breast cancer over the past ten years.

**Methods:**

This was a retrospective observational real-world study. The data were obtained from the clinical research database of three hospitals affiliated with Taipei Medical University. Patients with breast cancer who initiated their first chemotherapy regimen between January 1, 2011, and December 31, 2020, were identified by the ICD codes and their use of filgrastim or pegfilgrastim was identified by the Anatomical Therapeutic Chemical codes. Whether and how G-CSF was prescribed during the study patients’ first chemotherapy regimen was examined, and the annual change in the total number of short- and long-acting G-CSFs prescribed to the study patients from 2011 to 2020 was analyzed.

**Results:**

Among the 2,444 patients who were prescribed at least one of the examined 15 breast cancer chemotherapy drugs, 1,414 did not use any G-CSFs during their first chemotherapy regimen while 145 used G-CSFs for primary prophylaxis and 185 for treatment. Among the patients receiving high FN risk regimens, only 8.6% used G-CSF for primary prophylaxis. The average (± SD) number of days for short-acting G-CSF use was 2.3 (± 1.5) days with a median of 2 days. In addition, it was found that there was a significant reduction in long-acting G-CSF use (p = 0.03) whereas the changes in short-acting G-CSF use over time were not significant (p = 0.50).

**Conclusions:**

Our study results show that G-CSFs are used for primary prophylaxis in a small percentage of patients with breast cancer and the duration of short-acting G-CSF use is relatively short. Considering the significant clinical and economic impact of FN, it is hoped that the prescription patterns of G-CSFs observed can provide an important reference for future clinical practice and reimbursement policy.

## Introduction

Breast cancer is a common cancer worldwide, and there were more than 2.3 million new cases of female breast cancer in 2020 [1]. In Taiwan, breast cancer is the most common malignant tumor among women, with 14,856 women newly diagnosed with breast cancer in 2019 [2]. Treatment modalities of breast cancer include surgery, radiation, and drug therapies including myelosuppressive chemotherapy, hormonal therapy, immunotherapy, and targeted therapy [3]. Myelosuppressive chemotherapy is essential and effective but carries a potential risk of febrile neutropenia (FN) [4, 5].

FN is defined as an oral temperature of >38.3°C or two consecutive readings of >38.0°C for 2 hours and an absolute neutrophil count (ANC) either expected to fall below or already lower than 0.5 x 10^9^/L [6]. It is a potentially dose-limiting toxicity that can occur in patients with breast cancer who are receiving myelosuppressive chemotherapy, and it may cause prolonged hospitalization and is often associated with greater morbidity, mortality, and costs [7, 8].

Granulocyte colony-stimulating factor (G-CSF) is a hematopoietic colony-stimulating factor that stimulates the production, maturation, and activation of neutrophils to increase both their migration and cytotoxicity [9]. There are two types of G-CSFs: short-acting G-CSFs are administered once daily while long-acting G-CSFs are administered once per chemotherapy cycle [10]. Clinical practice guidelines have recommended prophylaxis with G-CSFs to reduce the incidence of FN in patients receiving chemotherapy. G-CSFs used for primary prophylaxis start with the first cycle of chemotherapy and continue through subsequent cycles while secondary prophylaxis with G-CSFs is given to patients who experienced neutropenia or FN during a prior cycle of chemotherapy [6, 11]. As recommended by the American Society of Clinical Oncology (ASCO) [11], the National Comprehensive Cancer Network (NCCN) [12], the European Society for Medical Oncology (ESMO) [6], and the European Organization for Research and Treatment of Cancer (EORTC) [13], primary prophylaxis with G-CSFs should be used for patients receiving chemotherapy regimens that are associated with a high risk of developing FN (>20%). For regimens with a low risk of FN (<10%), routine use of prophylaxis with G-CSFs is not recommended.

Until now, little research has studied the use of G-CSFs in patients with breast cancer in Taiwan. An exception was a study conducted in 2010 that found a chemotherapy regimen with a high risk of FN to be a risk factor for severe neutropenic events and also reported the duration of prophylactic use of short-acting G-CSFs. However, the study period was only one year, and long-acting G-CSFs were not examined [14]. In the present study, one of our aims was to examine the change in the use of long-acting and short-acting G-CSFs in breast cancer patients with myelosuppressive chemotherapy in Taiwan over the past ten years. In addition, we examined the use of G-CSFs for primary prophylaxis in Taiwan and see whether it follows the guidelines. The study findings will serve as an important reference for future clinical practice as well as future revisions of the reimbursement policies related to the long-acting and short-acting G-CSFs.

## Methods

### Study design

This was a retrospective observational real-world study. The study was approved by the Taipei Medical University-Joint Institutional Review Board (approval number: N202109004). As the study data was de-identified, informed consent was waived.

### Data source

The data used in this study came from the Taipei Medical University Clinical Research Database (TMUCRD). The database comprises the electronic medical records of the three affiliated hospitals of Taipei Medical University (i.e., Taipei Medical University Hospital, Wanfang Hospital, and Shuang Ho Hospital). It contains structured data (e.g., patients’ basic information, cause of death, medical information, cancer registry, laboratory test results, treatment procedures, surgeries, and medications) and unstructured data (e.g., physician notes, pathology reports, image examinations, and radiology reports). The TMUCRD started data collection in 1998, and by 2020 it had accumulated medical information for about 3.8 million patients in Taiwan. The data obtained for analysis were all de-identified.

### Study population

Patients who had one inpatient diagnosis or two outpatient diagnoses of breast cancer within a 365-day period [15–19] and then initiated their first chemotherapy regimen between January 1, 2011 and December 31, 2020 were identified by the International Classification of Diseases 9th/10th Revision Clinical Modification codes (ICD-9-CM 174, 175; ICD-10-CM C50, D05, C79.81). In addition, patients must have received regimens containing at least one of the 15 breast cancer chemotherapy drugs of interest and completed at least two cycles of the chemotherapy regimen. The 15 drugs were those listed in the EORTC and/or NCCN clinical practice guidelines [12, 13] that were available at the study hospitals (see Table 1).

**Table 1:** The 15 breast cancer chemotherapy drugs of interest.

Capecitabine	Doxorubicin	Paclitaxel
Carboplatin	Epirubicin	Pertuzumab
Cisplatin	Fluorouracil	Trastuzumab
Cyclophosphamide	Methotrexate	Trastuzumab emtansine
Docetaxel	Mitoxantrone	Vinorelbine

Exclusion criteria included HIV patients, patients who had received radiation therapy, and patients with hematopoietic stem cell transplants. In addition, patients younger than 20 years old at the first diagnosis of breast cancer were excluded.

### Chemotherapy regimens

The chemotherapy regimens examined in this study were the 15 selected chemotherapy drugs used as monotherapy or in various combinations. These regimens were determined mainly based on the EORTC clinical practice guidelines, supplemented by the NCCN guidelines [12, 13]. The regimens were classified into high (>20%), intermediate (10–20%), or low (<10%) risk of FN per the guidelines [12, 13]. Patients were categorized as having a high, intermediate, or low FN risk based on the chemotherapy regimen prescribed.

### Febrile neutropenia

In order to assess when the G-CSFs were being used for treatment rather than prophylaxis, it was necessary to define when FN had occurred. We identified the presence of FN using both a broad and a narrow definition. The broad definition is as follows: (1) body temperature >38°C and an ANC of <0.5 x 10^9^/L occurred within 7 days, or (2) body temperature >38°C and a diagnosis of neutropenia (ICD-9-CM 288.0x; ICD-10-CM D70.x) occurred within 7 days. The narrow definition is as follows: (1) having both diagnoses of fever (ICD-9-CM 780.6/ICD-10-CM R50.2, R50.9, R50.81-R50.84, R68.0, R68.83) [20] and neutropenia within 7 days, or (2) having both diagnoses of neutropenia and infection [21] within 7 days.

### G-CSF use

We examined whether the study patients were prescribed G-CSF during their first chemotherapy regimen and whether G-CSF was used for primary prophylaxis, treatment, or neither. The use of short-acting G-CSF (filgrastim) and long-acting G-CSF (pegfilgrastim) was identified by the Anatomical Therapeutic Chemical (ATC) code in TMUCRD. Primary prophylaxis was defined as the use of G-CSF within 7 days following the last dose of chemotherapy in the first cycle of the first chemotherapy regimen [22, 23]; the use of G-CSF was considered to be for treatment purposes if it was used when the patient had FN during the first chemotherapy regimen [14].

### Statistical analysis

The primary objective of this study was to examine whether and how G-CSF was prescribed for FN prophylaxis or treatment during the study patients’ first chemotherapy regimen. In addition, the use of G-CSFs for primary prevention was analyzed to see whether it adhered to the guidelines’ recommendations that primary prophylaxis with G-CSFs should be used for patients receiving chemotherapy regimens with a high risk of developing FN (>20%) [12, 13]. Moreover, the duration of the use of short-acting G-CSF was examined and divided into three groups (1 to 3 days, 4 to 6 days, ≥7 days). We also performed a logistic regression analysis to examine the association between the duration of short-acting G-CSF use and FN occurrence, adjusting for age and the FN risk level of chemotherapy regimens (high, intermediate, low). The secondary objective was to analyze the annual change in the total number of short- and long-acting G-CSFs used in the first chemotherapy regimen in the study patients from 2011 to 2020. The temporal changes in the use of short- and long-acting G-CSFs (i.e., prescription volume divided by the number of breast cancer patients receiving chemotherapy drugs examined) over the study period were assessed by linear regression analysis with the year being the independent variable.

Data were summarized using descriptive statistics. All statistical analyses were performed using SAS statistical software version 9.4 (SAS Institute). A P value less than 0.05 was considered statistically significant.

## Results

### Study patients

From January 1, 2011 to December 31, 2020, 6,104 patients with breast cancer who met the inclusion criteria were identified. Among them, 2,622 were prescribed at least one of the 15 breast cancer chemotherapy drugs under examination. After applying the exclusion criteria, 2,444 patients were included in the analysis.

### First chemotherapy regimens

Among the 2,444 patients, 1,840 (75.3%) patients were prescribed one of the examined regimens as their first chemotherapy regimen, with approximately 39.3% classified as high risk (Table 2).

**Table 2:** Patients by the classification of FN risks of the first chemotherapy regimens (N = 1,840).

FN risk	Patients (n)	(%)
High risk (>20% risk)	724	39.3%
Intermediate risk (10–20% risk)	588	32.0%
Low risk (<10% risk)	528	28.7%

### G-CSF use during the first chemotherapy regimen

As shown in Table 3 (the total percentage may be over 100% because a patient may have used G-CSF for both primary prophylaxis and treatment), using the broad FN definition, among the 2,444 patients, 1,414 did not use any G-CSF during their first chemotherapy regimen while 145 used G-CSFs for primary prophylaxis and 185 for treatment. When the narrow FN definition was applied, 150 patients used G-CSFs for treatment. In addition, among the 724 patients receiving high FN risk regimens, only 62 patients (8.6%) used G-CSF for primary prophylaxis (Table 4).

**Table 3:** G-CSF use during first chemotherapy regimens.

G-CSFs use	FN broad definition	FN narrow definition
	n (%)	n (%)
Primary prophylaxis	145 (5.9%)	145 (5.9%)
Treatment	185 (7.6%)	150 (6.1%)
Used neither for primary prophylaxis nor treatment	722 (29.5%)	753 (30.8%)
No use	1,414 (57.9%)	1,414 (57.9%)

**Table 4:** Primary prophylaxis of G-CSFs (N = 1,840).

FN risk	Patients with primary prophylaxis n (%)	Patients without primary prophylaxis n (%)	Total
High risk (>20% risk)	62 (8.6%)	662 (91.4%)	724 (100%)
Intermediate risk (10–20% risk)	21 (3.6%)	567 (96.4%)	588 (100%)
Low risk (<10% risk)	20 (3.8%)	508 (96.2%)	528 (100%)

In the analysis of the duration (i.e., the number of consecutive days) of the use of short-acting G-CSF, 88% of the prescriptions were for 1 to 3 days, 10.3% for 4 to 6 days, and 1.7% for 7 or more days. The average (± standard deviation) number of days for short-acting G-CSF use was 2.3 (± 1.5) days with a median of 2 days. The logistic regression results showed that the duration of short-acting G-CSF use was significantly associated with FN occurrence; a one-day increase in G-CSF use decreased the odds of FN by 33% (P < 0.0001).

### Change in G-CSF use over time

**Fig 1:**
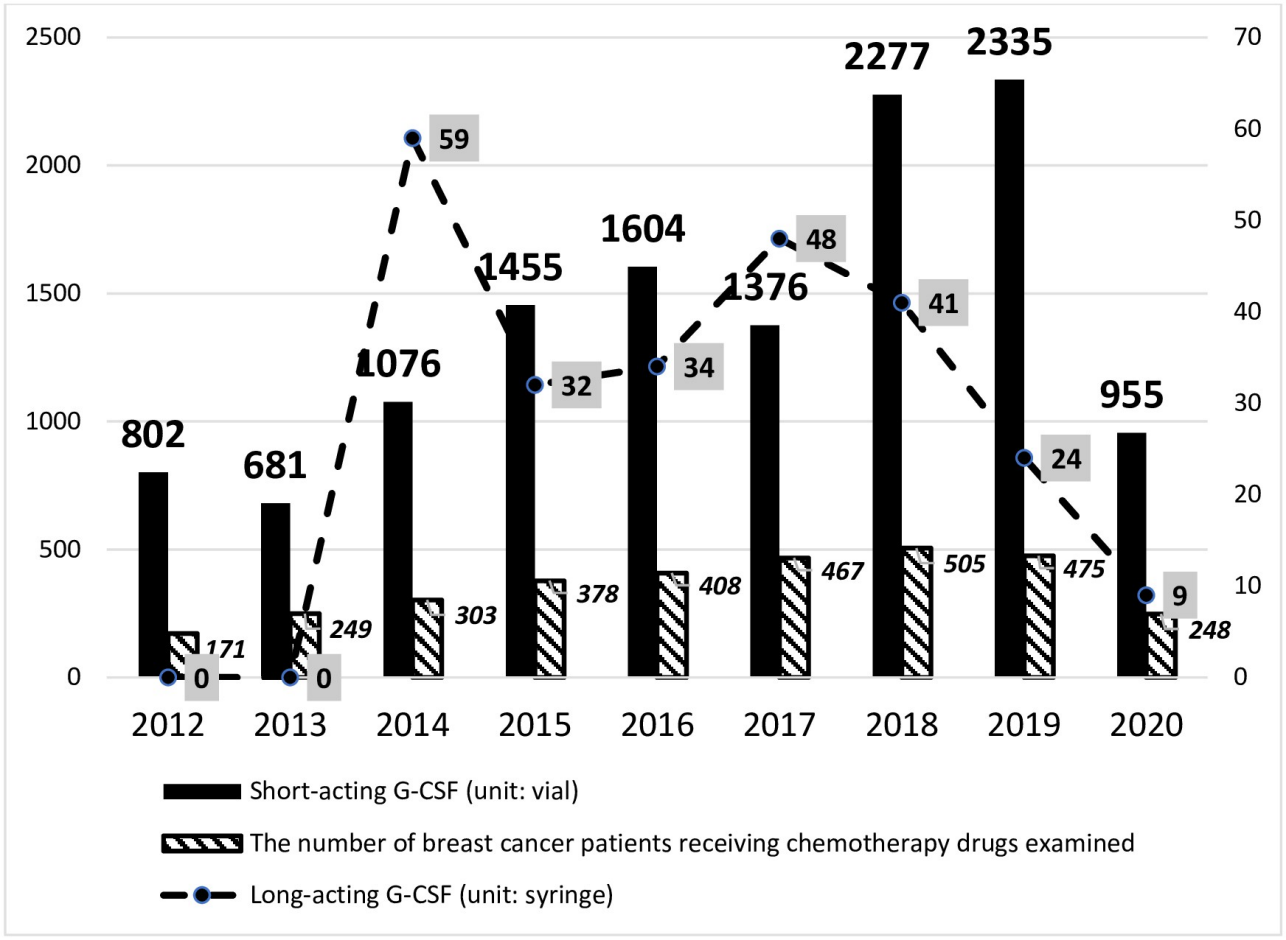
Annual use of long- and short-acting G-CSFs during the first regimen.

The total number of short- and long-acting G-CSFs used during the first chemotherapy regimen in 2,444 patients from 2011 to 2020 was analyzed, and the results are shown in Fig 1. By visual inspection, there was a steady increase in the use of short-acting G-CSF over the years while the use of long-acting G-CSF increased more briefly from 2013 to 2014 and then generally decreased after that. In 2020, the long-acting G-CSF continued its steady decline while the use of short-acting G-CSF also abruptly decreased. The regression analysis results showed that during the study period, there was a significant reduction in long-acting G-CSF use (β = - 0.02, p = 0.03) whereas the changes in short-acting G-CSF use over time were not significant (β = 0.07, p = 0.50). The changes in the prescription volume divided by the number of breast cancer patients receiving chemotherapy drugs examined over time can also be seen in S1 Fig.

## Discussion

In our study, only 8.6% of breast cancer patients receiving high FN risk regimens followed the guidelines’ recommended use of G-CSFs for primary prophylaxis. The observed low rate is likely due to the restrictions of reimbursement for G-CSFs under Taiwan’s National Health Insurance, which limits their use in solid tumors to treatment and secondary prophylaxis. Previous studies have reported that patients receiving high FN risk regimens who were given G-CSF as primary prophylaxis were less likely to have neutropenia-related dose delays and FN incidence compared to those who used it as secondary prophylaxis [24, 25]. Future research with a larger sample size is needed to investigate the actual benefits of using G-CSF for primary prophylaxis versus secondary prophylaxis in Taiwan.

Our study results found that the majority of short-acting G-CSFs were prescribed for a duration of 1 to 3 days with a mean (±S.D.) of 2.3 (± 1.5) days whereas in clinical trials, the required duration of short-acting G-CSF therapy was mostly 10 to 14 days [26]. We also found that a longer duration of short-acting G-CSF use is associated with less FN risk. Similarly, previous studies have reported that prophylaxis with short-acting G-CSFs for more than 7 days may be associated with a lower incidence of FN [27, 28]. In a previous study conducted in Taiwan in 2010, the duration of G-CSF use was also relatively short, with a mean of 4.9 and 3.7 days for primary and secondary prophylaxis, respectively [14]. The effectiveness of preventing FN may be compromised if short-acting G-CSFs are used for a suboptimal duration. The clinical consequences of a persistently short, and perhaps even decreasing, duration of G-CSF use in Taiwan need to be further investigated.

Our study also analyzed trends over time in the use of long- and short-acting G-CSFs in patients with breast cancer. The observed abrupt reduction in both long- and short-acting G-CSF prescriptions in 2020 was likely due to the outbreak of Covid-19 in Taiwan. In addition, since the dramatic increase in prescriptions for long-acting G-CSF when it first became available in the study hospitals in 2014, its use has generally decreased, which may have resulted in part from its stricter reimbursement restrictions compared to short-acting G-CSFs. According to NCCN guidelines, the use of long-acting G-CSFs is recommended for chemotherapy regimens given every 2 or 3 weeks [12]. A recent systematic review reported that in a variety of non-myeloid malignancies, prophylactic use of pegfilgrastim every 2 weeks was as safe and efficacious in reducing FN risk as filgrastim [29]. Previous studies have also found pegfilgrastim to be as effective as filgrastim for risk reduction of neutropenia-related or all-cause hospitalization [30] and duration of grade 4 neutropenia [31]. Moreover, compared to daily administration of filgrastim, single administration of pegfilgrastim had a lower rate of severe neutropenia, dose reduction, and treatment delay [32, 33].

The observed rate of using G-CSFs for primary prophylaxis in our study is much lower than that reported in studies conducted in Europe and America [34, 35]. For example, a prospective observational study in Spain showed that 60.6% of the breast cancer patients receiving high FN risk regimens initiated G-CSF from the first chemotherapy cycle [36]. The adherence rate to guidelines’ recommended primary prophylaxis in breast cancer patients receiving high-FN-risk chemotherapy cycles in Germany was 85.6% and 85.1% in 2006 and 2014–2015, respectively [37, 38]. The rate was similar in the Netherlands (88.3%) [39] while a lower rate was reported in the United States (48.5%) [40] and Puerto Rico (38.2%) [23]. Until now, little research has examined this issue in Asian countries.

Our study has several limitations. First, the three study hospitals belong to the same medical group and are all located in northern Taiwan, so the results may not be generalizable to other regions or hospitals. Second, database studies have inherent limitations, including potential data coding errors and missing data. Third, the chemotherapy drugs and G-CSFs prescribed in other hospitals could not be captured in the study database; nevertheless, it is uncommon for patients on chemotherapy to be treated in multiple hospitals at the same time.

## Conclusions

Our study results show that the proportion of breast cancer patients, including those receiving high-risk regimens, who are using G-CSFs for primary prophylaxis is low. In addition, the duration of use of a short-acting G-CSF is relatively short. Considering the significant clinical and economic impact of FN, it is hoped that the prescription patterns of G-CSFs observed can provide an important reference for future clinical practice and reimbursement policies.

## Supporting information

S1 Fig Temporal changes in the prescription volume divided by the number of breast cancer patients receiving chemotherapy drugs examined. (DOCX)
