# Supplementary material for: Prescription patterns of granulocyte colony–stimulating factors in patients with breast cancer: A real-world study
Source: PLoS One. 2023 Jul 17;18(7):e0288642. doi: 10.1371/journal.pone.0288642 (PMC10351717; doi:10.1371/journal.pone.0288642)
Supplement: S1 Fig — (DOCX) [file pone.0288642.s001.docx]

**Supplement 1**

Temporal changes in the prescription volume divided by the number of breast cancer patients receiving chemotherapy drugs examined
